# Small-molecule Bcl-2 antagonists as targeted therapy in oncology

**DOI:** 10.3747/co.v15i6.392

**Published:** 2008-12

**Authors:** M.R. Warr, G.C. Shore

**Keywords:** Apoptosis, Bcl-2

## Abstract

Dynamic protein–protein interactions between proapoptotic and pro-survival Bcl-2 family members regulate outer-mitochondrial membrane permeabilization and cytochrome c release, key events in the path to apoptosis. Their relative levels often dictate the fate of a cell following an apoptotic stimulus. However, in cancer cells, the pro-survival Bcl-2 family members are frequently upregulated, thereby creating a constitutive block to apoptosis and resulting in continued cell survival under conditions that normally result in cell death. Because many chemotherapeutics used to treat cancer also trigger apoptosis, this upregulation of pro-survival members also contributes to resistance to conventional cancer therapies. Strategies that inactivate pro-survival Bcl-2 family members therefore suggest a means by which this downstream block in apoptosis can be alleviated, resulting in the selective killing of malignant cells. Here, we outline the progress of three small-molecule Bcl-2 antagonists that have advanced into clinical evaluation.

## 1. INTRODUCTION

Apoptosis evolved in metazoans as a means to maintain tissue homeostasis by eliminating unwanted or damaged cells. By removing cells that have accumulated genetic mutations that subvert normal growth control, apoptosis forms a protective barrier against oncogenic transformation. In fact, evasion of apoptosis is considered to be a hallmark of all cancers, because cancer cells manifest stress signals such as transforming oncogenes that trigger apoptosis and should result in their self-elimination and corresponding abrogation in tumour formation 1. Thus, for a cell to become malignant, it must first bypass the apoptotic machinery. This task can be accomplished through either inactivation of apoptosis-inducing genes or activation of inhibitors of apoptosis. Because many chemotherapeutic drugs used to treat cancer also trigger apoptosis, evasion of apoptosis may also contribute to the resistance encountered in conventional cancer therapies.

## 2. THE BCL-2 FAMILY

Many apoptotic signals, such as dna damage and oncogene activation, initiate signalling cascades that converge on the permeabilization of the outer mitochondrial membrane (omm). This membrane permeabilization leads to the release of cytochrome c from the inter-membrane space, resulting in the activation of a family of aspartate-specific cysteine proteases, termed *caspases.* Activated caspases in turn cleave numerous cellular proteins, resulting in the morphology known as apoptosis and, ultimately, in cell destruction 2–4. These pathways are both executed and regulated by the Bcl-2 family of proteins, which comprise three groups, defined according to function and to Bcl-2 homology (bh) domain content 5–8:

- The proapoptotic effectors, including Bax and Bak, contain bh1, bh2, and bh3, and a C-terminal trans-membrane segment that selectively targets these proteins to the membranes of mitochondria and endoplasmic reticulum. Bax and Bak are present as inactive monomers under normal conditions; however, following a death signal, they can undergo homo- and hetero-oligomerization within the omm, causing omm permeabilization and the egress of cytochrome c from the inter-membrane space. They form an essential gateway for the mitochondrial apoptotic pathway: mice deficient in both Bax and Bak are resistant to all tested intrinsic apoptotic signals 9,10.
- The pro-survival members Bcl-2, Mcl-1, Bcl-xl, Bcl-w, and A1 have the same overall architecture as Bax and Bak, but with the exception of Mcl-1, they also contain a bh4 domain located toward their N-terminus.
- The last group, a large and diverse proapoptotic group within this family, contains only a single bh3 domain and includes Bim, Puma, Bid, Bad, Bik, Noxa, and others. They respond to death signals upstream of Bax and Bak, resulting in their activation or upregulation (or both). The active bh3-only members then facilitate transition of these signals to the downstream multi-domain members 7,11.

The outcome of cell death signalling pathways depends on a complex interplay involving physical interactions between the pro-survival and pro-death members. Structural studies have revealed that the bh3 domain adopts an alpha-helical conformation and that the bh3 domain of a proapoptotic member is able to bind to pro-survival members by occupying a hydrophobic pocket formed by the close proximity of their bh 1–3 domains 12,13.

**FIGURE 1:**
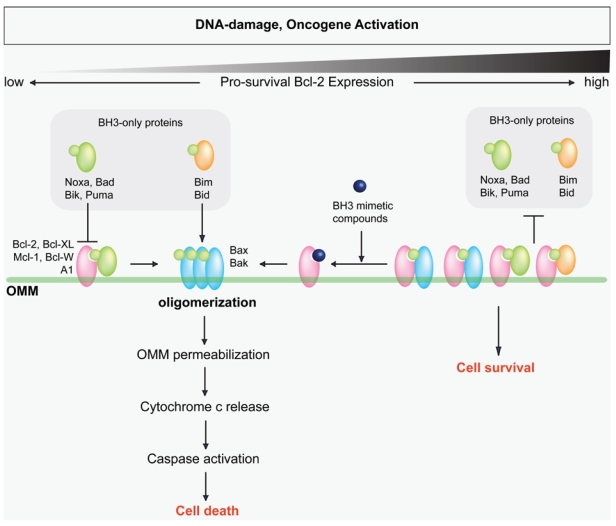
Regulation of mitochondrial apoptotic pathway by the Bcl-2 protein family. The schematic details how the Bcl-2 family integrates upstream death signals to Bax and Bak oligomerization, permeabilization of the outer mitochondrial membrane (omm), and release of cytochrome c. Overexpression of pro-survival Bcl-2 family members block Bax and Bak oligomerization, resulting in cell survival.

The bh3-only proteins have been proposed to form two distinct groups: those that sensitize cells to apoptosis by binding to anti-apoptotic Bcl-2 proteins (Puma, Bad, Noxa, and Bik), and those that in addition directly activate proapoptotic Bax or Bak (Bim and Bid) 14,15. In this model, apoptosis will not proceed unless sufficient activator bh3-only proteins are present, because sensitizer bh3-only proteins cannot independently activate Bax and Bak. However, whether bh3-only proteins can directly activate Bax and Bak, or whether an additional activation event is even needed for Bax and Bak oligomerization and cytochrome c release is currently under debate^14^,^16^–^18^. Similar controversy exists concerning how the pro-survival members inhibit Bax and Bak oligomerization; however, they likely both function to sequester bh3-only proteins and to antagonize Bak and Bax directly at the mitochondria (Figure 1) 14,16,19.

Because many apoptotic pathways are regulated and executed by the Bcl-2 family, the ratio between pro-survival Bcl-2 family members and proapoptotic members often dictates the fate of a cell following an apoptotic stimulus. In cancer cells, this balance is often altered and thus provides a means for cancer cells to evade apoptosis. The founding Bcl-2 family member, Bcl-2, was identified at the chromosomal breakpoint t14:18 in human follicular B-cell lymphoma, resulting in its upregulation. Since this discovery, many malignancies have been shown to overexpress Bcl-2 and other pro-survival family members 20,21. This overexpression results in a constitutive block to Bax and Bak oligomerization, thereby preventing translation of the upstream death signals to omm permeabilization (Figure 1). Strategies that target the functional bh3-binding groove of these pro-survival Bcl-2 family members in an attempt to reinstate an effective apoptosis pathway therefore hold significant therapeutic promise in oncology 22,23.

## 3. THE SMALL-MOLECULE BCL-2 ANTAGONISTS

Several small-molecule Bcl-2 antagonists have been developed and are under preclinical evaluation. Here, however, we focus on compounds that have advanced into clinical trials.

### 3.1 Obatoclax

A high throughput screen used to identify molecules that interrupt Bcl protein–protein interactions identified GX15-070 (obatoclax) 23,24. By fluorescence polarization assay, obatoclax has been reported to bind the bh3 groove of all pro-survival members with low micromolar affinity 25. However, obatoclax is particularly hydrophobic and therefore insoluble in strictly aqueous assays. The use of fluorescence polarization assays or similar binding approaches to determine the exact affinity of this compound for Bcl-2 family members may therefore not be possible 24.

Notably, obatoclax disrupted the protection afforded by Bcl-2, Mcl-1, Bcl-xl, and Bcl-w in a Bax and Bak reconstituted yeast system, and inhibited colony formation of Eμ–*myc* lymphoma cell lines overexpressing Bcl-2, Mcl-1, and A1, suggesting that it can target multiple pro-survival Bcl-2 family members 24. Obatoclax displayed single-agent activity against multiple cancer cell lines, including non-small-cell lung cancer (nsclc), multiple myeloma (mm), and patient-derived mantle-cell lymphoma, acute myelogenous leukemia (aml), and chronic lymphocytic leukemia (cll) cells 26–31. *In vivo* tumour regression was seen in xenograft models derived from prostate, cervical, colon, and mammary carcinoma cell lines 24. Furthermore, obatoclax displayed synergy with bortezomib in mantle-cell lymphoma and mm, with cisplatin in nsclc, with cytarabine in aml, and with fludarabine in cll 27–29,31.

Obatoclax-mediated apoptosis depends on Bax and Bak, as evidenced by loss of caspase 3 activation and dna fragmentation in mouse kidney epithelial cells doubly deficient in Bax and Bak 24. A number of groups have reported that obatoclax cytotoxicity correlates with the disruption of Mcl–Bak complexes as measured by chemical crosslinking in isolated mitochondria and immunoprecipitation 24,27,29–31, but disruption of Bcl-xl–Bak, Mcl-1–Bim, and Bcl-2–Bim complexes have also been noted 26,29,31. A substantial reduction in apoptotic cells was observed in mouse embryonic fibroblasts deficient in Bak and Bim, further suggesting a central role for Bak and Bim in obatoclax-mediated cytotoxicity^31^. Obatoclax is currently being investigated in phase i and ii clinical trials as both a single agent in cll, aml, cll, and Hodgkin lymphoma, and in combination with other antitumor agents in hematologic, lymphoid, and solid-tumour malignancies. (Details can be found at www.clinicaltrials.gov/ct2/results?term=obatoclax.)

### 3.2 ABT-263

Nuclear magnetic resonance screening was used to develop a small-molecule inhibitor of Bcl-xl. The resulting compound, ABT-737, binds with high affinity to Bcl-2, Bcl-xl, and Bcl-w (K_i_ ≤ 1 nmol/L), but exhibits minimal binding to Mcl-1 And A1 (K_i_ > 1 μmol/L)^32^. This behaviour is consistent with the agent’s ability to inhibit colony formation of Eμ–*myc* lymphoma cell lines overexpressing Bcl-2, but not Mcl-1 or A1^24^. ABT-737 showed single-agent activity against a variety of established solid tumour, hematopoietic, and lymphoid cancer cell lines, and against primary patient-derived follicular lymphoma, acute lymphoblastic leukemia (all), and mm cells 32–36. Furthermore, tumour regression with limited toxicity was seen in small-cell lung cancer, mm, and aml tumour xenografts 32,35,37. In addition, ABT-737 exhibited synergistic cytotoxicity with paclitaxel in nsclc cells, with etoposide in nsclc xenografts, and with bortezomib, melphalan, and dexamethasone in mm cells 32,33,35,37.

Mechanistically, ABT-737 has been shown to disrupt Bcl-2–Bim and Bcl-2–Bax complexes as demonstrated by immunoprecipitation 35,38. Furthermore, apoptosis induction was greatly diminished in Bak-, Bax-, and doubly-deficient mouse embryonic fibroblasts, suggesting that ABT-737–mediated cytotoxicity requires both Bak and Bax^35^. More recently, an orally available analog of ABT-737, ABT-263, which has a similar binding profile, has been created^39^ and is currently in early phase i and ii clinical trials as a single agent for a variety of hematologic, lymphoid, and solid-tumour malignancies. (Details can be found at www.clinicaltrials.gov/ct2/results?term=abt263.)

### 3.3 (–)-Gossypol

Gossypol is a natural product isolated from cottonseeds that has been studied previously for a variety of therapeutic uses. More recently, it was found that the (–) enantiomer of gossypol (AT-101) binds with nanomolar affinity to Bcl-2 (320 nmol/L), Bcl-xl (480 nmol/L), and Mcl-1 (180 nmol/L) 40. Single-agent activity of (–)-gossypol has been shown in a variety of cancer cell lines, including cll, prostate and colon carcinoma, and patient-derived mm and diffuse large-cell lymphoma cells^41^–^46^. Furthermore, synergy was seen with dexamethasone in mm and with docetaxel in prostate cancer cell lines^42^,^43^. In addition, (–)-gossypol was shown to be effective with cyclophosphamide and rituximab in a B-cell lymphoma xenograft model^47^.

Mechanistically, gossypol has been shown to disrupt Bcl-xl–Bim and Bcl-xl–Bax complexes^44^. However, gossypol has recently been shown to induce apoptosis in a Bax- and Bak-independent manner by converting Bcl-2 into a proapoptotic molecule^48^. Notably, the conversion was accomplished using a mixture of gossypol enantiomers, suggesting that tests should be repeated with (–)-gossypol alone. Further work needs to be done to ascertain gossypol’s primary mechanism of action. Currently, (–)-gossypol is being evaluated in phase i and ii clinical trials as a single agent in B-cell malignancies and prostate cancer, and in combination with other antitumor agents in a variety of hematologic, lymphoid, and solid-tumour malignancies. (Details can be found at clinicaltrials.gov/ct2/ results?term=gossypol.)

## 4. MCL-1: A CRITICAL TARGET IN CERTAIN MALIGNANCIES

Genetic mouse models have demonstrated that pro-survival Bcl-2 family members play critical, non-redundant roles in the development and maintenance of normal tissues 49. In addition to varied expression, this property of Bcl-2 proteins may be at least partly explained by the existence of considerable bh3-dependent binding preferences between pro-survival Bcl-2 family members and their proapoptotic partners^14^,^19^,^50^. For example, Bim and Puma have been shown to interact with high affinity with all pro-survival members, but Noxa and Bad interact selectively only with Mcl-1 and A1, and with Bcl-2, Bcl-xl, and Bcl-w respectively^14^,^50^.

The ABT-737 molecule, which does not bind Mcl-1 and A1, and which therefore has a binding profile similar to that of Bad, exhibits single-agent cytotoxicity in select tumour models as described. However, ABT-737 encounters marked resistance under conditions in which Mcl-1 is elevated^24^,^38^,^51^,^52^. Correspondingly, targeted downregulation of Mcl-1 by short interfering rna sensitizes cells resistant to ABT-737 in a number of malignancies 24,35,52–55. This interference correlates with the inability of ABT-737 to effectively bind and neutralize Mcl-1 32. Therefore, to bypass cancer cell survival with a Bcl-2 antagonist, neutralizing Mcl-1 appears to be critical in certain tumour environments.

A variety of means could be employed to combat resistance encountered by Mcl-1. These include the use of a high-affinity pan–Bcl-2 inhibitor (although this approach may increase toxicity), combinational use of multiple Bcl-2 antagonists, or subsequent treatment with agents that diminish Mcl-1 levels. In fact, obatoclax (which has been shown to displace proapoptotic members from Mcl-1) and etoposide, carboplatin, sorafenib, and seliciclib (all of which have been shown to reduce Mcl-1 levels) can synergize with ABT-737 to overcome Mcl-1–mediated resistance 24,52–55. Of course, use of a selective small-molecule inhibitor to inactivate Mcl-1 protein levels represents a considerably more direct way of targeting Mcl-1–mediated resistance, and therefore may allow for greater functional utility.

Resistance to ABT-737 mediated by Mcl-1 provides an example of the complexity involved in targeting pro-survival Bcl-2 family members in cancer. Furthermore, it ultimately suggests that the efficacy of small-molecule Bcl-2 antagonists in the clinic will depend on neutralizing the pertinent pro-survival member or members that are selectively sequestering proapoptotics needed to link an upstream death signal to downstream caspase activation.
